# Adult food choices in association with the local retail food environment and food access in resource-poor communities: a scoping review

**DOI:** 10.1186/s12889-023-15996-y

**Published:** 2023-06-06

**Authors:** Samukelisiwe S. Madlala, Jillian Hill, Ernesta Kunneke, Tatum Lopes, Mieke Faber

**Affiliations:** 1 Non-Communicable Diseases Research Unit, South African Medical Research Council, Cape Town, South Africa; 2 School of Public Health, Faculty of Community and Health Sciences, University of the Western Cape, Cape Town, South Africa; 3 Department of Dietetics and Nutrition, University of the Western Cape, Cape Town, South Africa; 4 Division of Chemical Pathology, Department of Pathology, Faculty of Medicine and Health Sciences, University of Stellenbosch, Tygerberg Hospital, Cape Town, South Africa; 5 Centre of Excellence for Nutrition, North-West University, Potchefstroom, South Africa

**Keywords:** Food environment, Adults, Food choice, Food access, Resource-poor

## Abstract

**Background:**

There is a growing body of research on local retail food environments globally in both urban and rural settings. Despite this, little research has been conducted on adult food choices, local retail environments, and healthy food access in resource-poor communities. The purpose of this study is therefore to provide an overview of the evidence on adult food choices (measured as dietary intake) in association with the local retail food environment and food access in resource-poor communities (defined as low-income communities and/or households).

**Methods:**

We searched nine databases for studies published from July 2005 to March 2022 and identified 2426 records in the primary and updated search. Observational studies, empirical and theoretical studies, focused on adults ≤ 65 years, published in English peer-reviewed journals, examining local retail food environments and food access, were included. Two independent reviewers screened identified articles using the selection criteria and data extraction form. Study characteristics and findings were summarized for all studies and relevant themes summarized for qualitative and mixed methods studies.

**Results:**

A total of 47 studies were included in this review. Most studies were cross sectional (93.6%) and conducted in the United States of America (70%). Nineteen (40.4%) studies assessed the association between food choice outcomes and local retail food environment exposures, and evidence on these associations are inconclusive. Associations of certain food choice outcomes with healthy food retail environments were positive for healthy foods (in 11 studies) and unhealthy foods (in 3 studies). Associations of certain food choice outcomes with unhealthy retail food environment exposures were positive for unhealthy foods in 1 study and negative for healthy foods in 3 studies. In 9 studies, some of the food choice outcomes were not associated with retail food environment exposures. A healthy food store type and lower food prices were found to be major facilitators for healthy food access in resource-poor communities, while cost and transportation were the main barriers.

**Conclusions:**

More research is needed on the local retail food environment in communities in low- and middle-income countries to develop better interventions to improve food choices and access to healthy foods in resource-poor communities.

**Supplementary Information:**

The online version contains supplementary material available at 10.1186/s12889-023-15996-y.

## Background

Globally, poor diet is a primary risk factor for death and disability [1] and is responsible for various types of malnutrition [2]. In 2016, > 1.9 billion adults (39%) worldwide were overweight and of these 650 million (13%) were obese [3]. On the other side of the spectrum, an estimated 768 million people (10%) worldwide were undernourished and 928 million people (12%) were severely food insecure in 2020 [4]. Poor food systems and unhealthy food environments contribute to the high global prevalence of poor nutritional status [2].

Food choices are influenced by the various physical, economic, political and socio-cultural environments in which people live [5, 6]. The collective of these environments are referred to as the food environment, which reflects the context in which people acquire, prepare and consume foods [5, 7]. According to Glanz and colleagues, local food environments can be categorized into the community nutrition environment, consumer nutrition environment, and organizational nutrition environment [8]. The community nutrition environment refers to number, type, location and accessibility to food stores in a community. The consumer nutrition environment refers to the availability of healthy food choices, price, promotion, quality and placement of food items [8]. The community and consumer nutrition environments combined are referred to as the retail food environment [9]. The retail food environment can therefore be described as accessibility to local food stores and markets, and the availability and affordability of healthy foods in these stores and markets [8].

The five dimensions of the food environment, also known as the dimensions of food access include availability, accessibility, affordability, acceptability and accommodation [10]. In the context of the food environment, availability refers to the density (presence) of different types of food stores within a specific area such as census tracts or buffer zones [10, 11]. Accessibility refers to (i) geographic location of the food stores, defined as proximity which can be measured as travel time and distance to stores [10, 11], and (ii) diversity or variety of different types of food stores, such as supermarkets and fast food (FF) restaurants [12]. Affordability refers to purchasing power and food prices, measured by store audits or price indices [10]. Acceptability refers to people's attitudes on the characteristics of their local food environment, it can be measured as people’s perception on quality of foods sold or as store audit food quality score [10]. Accommodation refers to how well the local retail food environment caters to residents' needs such as store operating hours and types of payment options offered to customers [10]. Perceptions on availability, accessibility affordability, acceptability and accommodation in the local retail food environment can also be measured [13].

Food choice is defined as the processes by which people consider, acquire, prepare, store, distribute, and consume foods and beverages [14]. Food choice is determined by individual and social factors, as well as physical and macro-level environments such as the food system [15]. Changes in the food environment due to changes in food supply and demand affect individuals’ food choices [16]. Food environments therefore affect diet quality and dietary habits, and ultimately impact diet-related health outcomes [17–19]. In their review paper, Story and colleagues’ reported that healthy retail food environments have been shown to be characterized by access to food stores such as supermarkets, grocery stores and farmers markets, and limited presence of FF restaurants in a community, and the availability of healthy affordable food products within stores [20]. A healthy food environment can lead to improved access to fruits and vegetables (FV), greater dietary diversity [21], and provision of healthier options of pre-packaged foods, prepared and readymade meals in different types of retail food stores [22].

The FAO defines food deserts as geographic areas where grocery stores, farmers markets and other healthy food providers are not located within a reasonable travelling distance of residents, restricting their access to healthy food [5]. Canadian studies described food swamps as geographic areas with access to retailers with healthy food options but also a large number of convenience stores, FF outlets and other outlets that sell predominantly unhealthy foods and beverages [23]. Access to healthy food is therefore restricted in food deserts, while unhealthy food is more readily available in food swamps. Food deserts or food swamps are most likely to occur in resource-poor areas [12, 23, 24]. In the United States of America (USA), a study on FF restaurants and convenience stores within close proximity to schools showed that that convenience stores and FF restaurants are most likely to be located in lower-income neighborhoods, and that convenience stores generally stock limited variety of foods, have high prices and stock foods of a lower quality [25]. Studies have shown that living in close proximity to FF restaurants [24] and greater access to convenience stores in comparison to supermarkets may reflect an unhealthy food environment [26].

Residing in a food desert has been associated with inadequate diets [27] and increased risk of obesity [28]. Resource-poor communities often lack access to healthy food such as fresh FV [29–31], and are more susceptible to poor nutrition and diet-related diseases because of their lack of access to healthy and affordable foods [32]. It has been reported that neighborhood deprivation is associated with inadequate dietary patterns [33], and that people with low socioeconomic status (SES) have low quality diets as they consume more energy-dense and nutrient-poor foods [32]. For the purposes of this scoping review the terms community and neighborhoods are used interchangeably.

Research on the food environment is rapidly growing and several systematic reviews on different aspects of the food environment have been published. To date, systematic reviews focused mostly on the relationship of the local food environment with dietary outcomes and nutritional status [10, 34–38], childhood overweight and obesity [26, 39–43], FF access in food environments [44, 45], food purchasing and food environment [46], community and consumer food environment and children’s diet [47–49], and the food environment in low- and middle-income countries [35, 50]. Despite the growing body of research, there is limited synthesis on the characteristics of the food environment that relate to food choices per se, particularly for adults residing in resource-poor communities [51, 52].

There is a greater need to understand the relationship between food environments and diets as government and policy makers are seeking interventions to combat the rise of obesity globally [6, 52]. Therefore, the aim of the scoping review is to provide an overview of the evidence on adult food choices in association with the local retail food environment and food access in resource-poor communities. The objectives of the scoping review are to 1) assess whether adult food choices are associated with the local retail food environment in resource-poor communities; and 2) determine the barriers and facilitators for healthy food access within the local retail food environment in resource-poor communities. Food choice in the context of this scoping review refers to dietary and food intake and pertains to diet scores, diet quality, FV intake, food group intake, salty, fatty, and sugary foods and SSB intake. We defined resource-poor communities as low-income communities/neighborhoods, disadvantaged communities/neighborhoods, and/or low-income/low socioeconomic position (SEP) households/individuals.

### Methods

#### Study design

A scoping review of the literature on adult food choices in association with the local retail food environment and food access in resource-poor communities was conducted, following the scoping review framework outlined by Arksey and O’Malley [53]. A scoping review was conducted to scope the body of literature and to identify knowledge gaps on the topic. The Preferred Reporting Items for Systematic Reviews and Meta-Analyses (PRISMA) extension for scoping reviews (PRISMA-ScR) [54] was used to guide the review process (see Additional file 1).

### Registration and protocol

The protocol for this scoping review was registered on the Open Science Framework on 9 September 2020 (https://osf.io/shf93), and is available online [55].

### Search strategy

The population, concept and context (PCC) framework was applied to inform the search strategy [56]**.** A systematic literature search of eight multidisciplinary databases and a research platform namely, PubMed/MEDLINE, CINAHL, Green FILE, PsycARTICLES, Social Science Research Network, Scopus, Science Direct, Web of Science and EBSCOhost was performed. Search keywords or medical subject headings (MeSH) were used. Details on the keywords and Mesh terms are described in the protocol [55]. The Boolean (AND, OR) method was used to combine search terms. The original search strategy was developed in PubMed/Medline and was adapted to the other databases (detailed search strategies are listed in Additional file 2). The main concepts searched were based on diet/food choice AND adult AND local retail food environment OR community OR consumer food environments AND resource poor AND food access AND store type. Date restrictions in the original search were set between 2005 and January 2021. The search was updated to include studies published between February 2021 and March 2022.

### Inclusion criteria

This review included observational studies (cohort, cross-sectional, case–control and ecological studies) examining the association between adult food choices (outcome) and the local retail food environment and food access (exposures) in resource-poor communities, empirical and theoretical studies, studies including adults 18 – 65 years old, studies on the retail food environment, which includes the community and the consumer food environment, studies on food access, food choices and diets of adults in resource-poor communities and English peer-reviewed journal articles from July 2005 to March 2022 [55].

### Exclusion criteria

Excluded studies were experimental studies (randomized control trials), systematic reviews, and meta-analysis, research not reported in peer-reviewed journals, studies examining the organizational food environment (home, school, and work) and information environment (television advertising), studies on children, pregnant women, and the elderly, studies that only focus on the food environment and nutritional status, studies focusing on indirect measures of diet, such as food purchasing or the number of food store visits, research papers not written in English, and papers published before July 2005 [55]. After conducting the pilot study ‘Other’ and ‘National study’ were added as the eighth and nineth exclusion reason. ‘Other’ refers to papers that were irrelevant to the study but could not be classified under any of the listed exclusion criteria. ‘National study’ refers to studies for which results were reported at national level, with no distinction between groups or settings of different socio-economic status. After conducting the first round of full text article screening two more exclusion reasons were added: not reporting association between adult food choices and local retail food environment, and not reporting barriers and facilitators for healthy food access in resource-poor communities.

### Screening

**Fig. 1:**
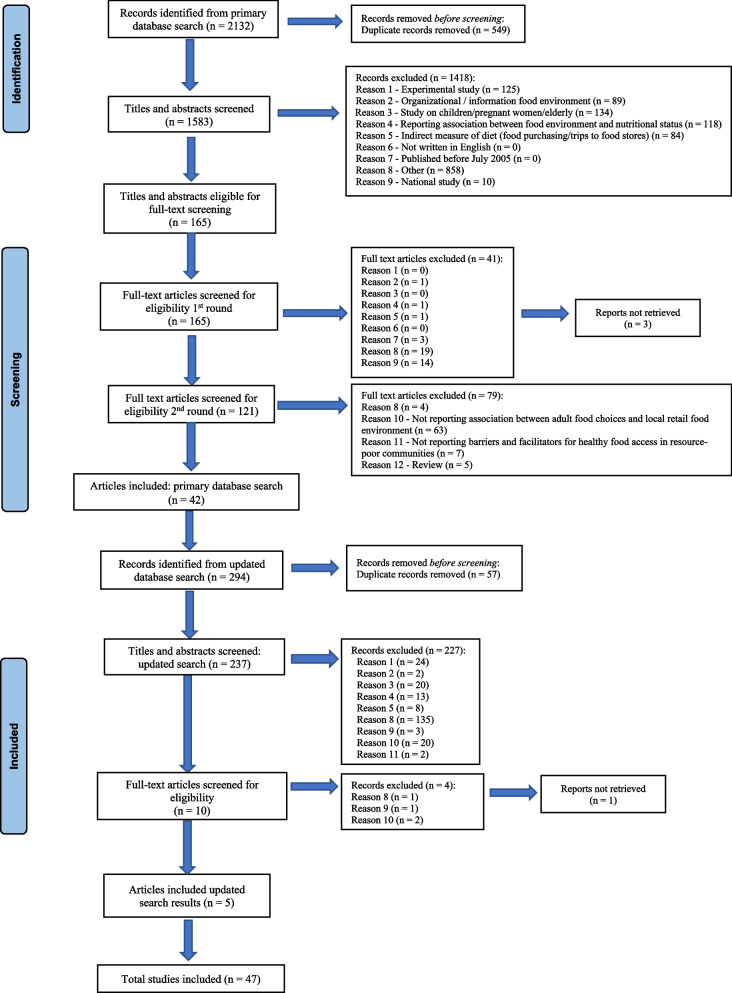
PRISMA flow diagram of scoping review

The primary database search was done for studies published between July 2005 and January 2021, which was updated through a second search to include studies published from February 2021 to March 2022 (see Fig. 1). Studies identified were exported to EndNoteX9 library, and duplicates were identified and removed. The primary database search identified 2132 studies, and after duplicates were removed 1583 records remained. Two reviewers (SSM and TL) independently screened the title and abstracts (TIABS). Of the 1583 TIABS screened, 165 were identified as eligible for full-text screening. The two reviewers independently read the full-text articles to determine whether they meet the eligibility criteria. Full-text screening for the primary database search was done in two rounds. In the first round of full-text screening, 165 articles were screened and 121 articles were deemed eligible. In the second round of screening, 121 articles were screened and 42 articles primary database search articles were eligible for inclusion in the scoping review. In the updated database search, 294 records were identified. After removing duplicates, 237 TIABS were screened. After screening TIABS, 10 articles were eligible for full-text screening. After full-text screening of the updated search results, five studies were deemed eligible for inclusion. Therefore, a total of 47 studies (42 articles from the primary search and five from the updated search) were included. Both TIABs and full-text article screening were performed on the Rayyan Qatar Computing Research Institute (QCRI) systematic reviews web application [57].

### Data extraction

A data collection form based on the framework of Arksey and O’Malley [53] was used to obtain the following information from each study: name of authors, title, year of publication, aim/objective of the study, study area, study setting, study participants, sampling method, study design, data collection, measurement tools, data analysis, reported outcomes, most relevant findings, facilitators and barriers (see Additional file 3). The data extraction form was piloted on a sub-sample of 17 articles to ensure the form captures relevant data and ensures consistency between reviewers. The data extraction form was revised to improve capturing of study methods employed in the research. Interrater agreement was high (78%). The percent agreement for two raters was calculated as the number of agreements (full text articles included and excluded by both raters) divided by the sum of the number of agreements and the number of disagreements (conflicts) multiplied by 100 [58]. The calculation was as follows: 137 / (137 + 38) × 100 = 78. Disagreements were resolved through discussion between the two reviewers.

Study characteristics and findings were summarized for all studies, and relevant themes summarized for qualitative and mixed methods studies [53, 59]. We synthesized identified studies by dividing them into two groups 1) studies on the association between food choice and the local retail environment; and 2) studies reporting barrier and facilitators to healthy food access. Barriers and facilitators were further categorized by study design into quantitative, mixed method, and qualitative studies. Qualitative studies and mixed methods reporting relevant qualitative data were grouped together in tables. Quantitative data from mixed method studies were grouped in tables with quantitative data from non-mixed method studies.

## Results

### Overview of studies included

**Fig. 2:**
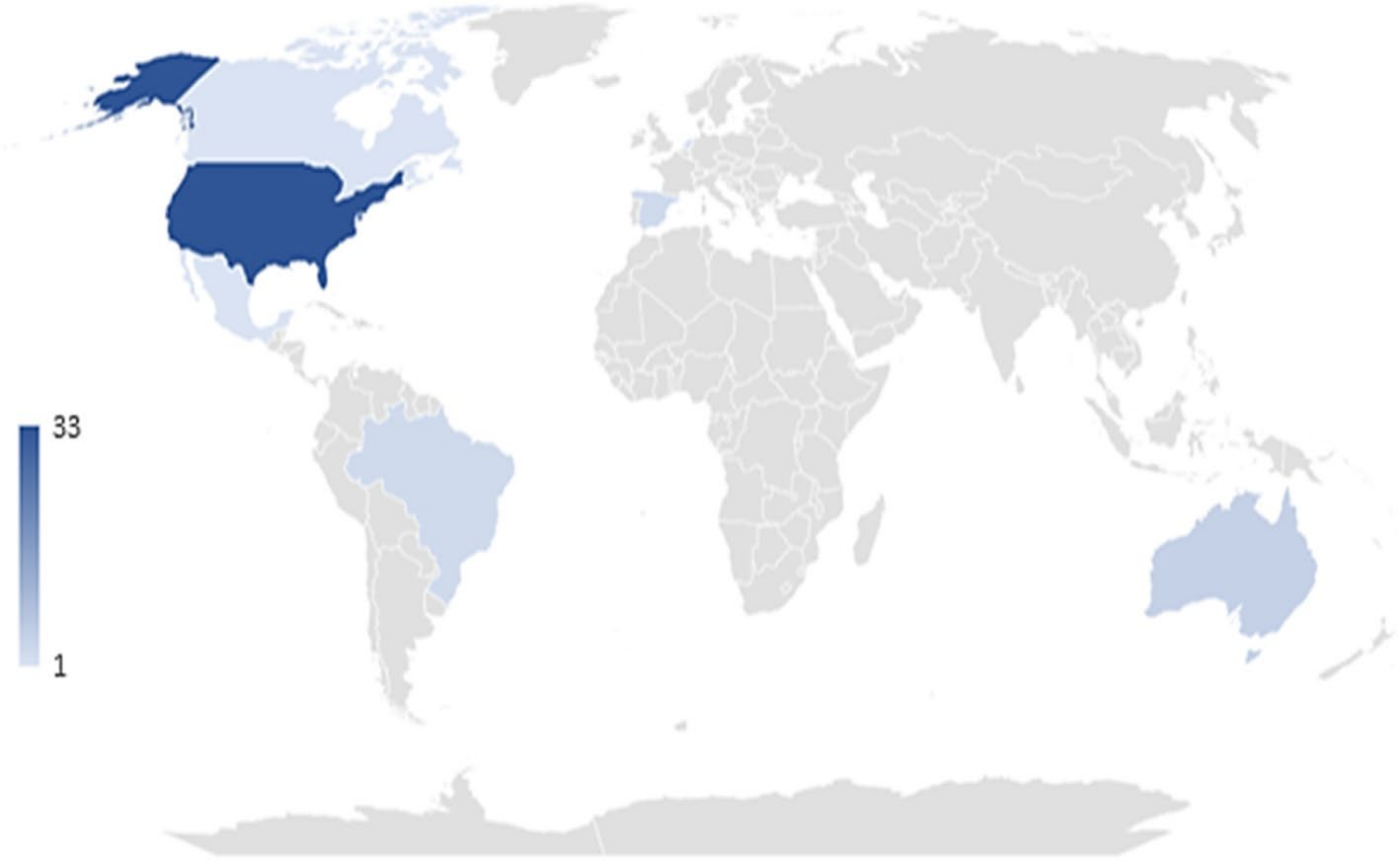
Map showing countries of studies included in the scoping review

Forty-seven articles, published between 2006 and 2021, were eligible for inclusion in this review (see Table 1). Most (93.6%) of the studies were cross-sectional in design, except for two cohort studies and one ecological study. To examine the associations between local retail food environment and food choice and to describe barriers and facilitators to healthy food access in the local retail food environment, 23 studies used quantitative methods, nine used qualitative methods, and 15 used mixed methods. Approximately 70% (*n* = 33) of studies were conducted in the USA, five in Australia, three in Brazil, three in Spain, one in Mexico, one in Netherlands and one in Canada (see Fig. 2). In total, 76.6% (*n* = 36) of the studies were conducted in urban settings and 14.9% (*n* = 7) in rural settings. The age of the participants in the studies ranged from 18 to 84 years. Studies were included if the mean age of participants was within the study inclusion criteria. Terms used to describe resource-poor communities included low income, disadvantaged neighborhoods, and low SEP.

**Table 1:** Characteristics of included studies (*n* = 47)

**Author (Year)**	**Location/ Country**	**Setting**	**Sample (n)**	**Study design**	**Methods**				**Food Environment Dimensions**				
					GIS	Store audit/Survey	Dietary questionnaire	Qualitative	Availability	Price	Accessibility	Affordability	Quality
Alkon et al. [60]	Oakland & Chicago, USA	Urban	*N* = 27 adults (24 women & 3 men)	Cross-sectional: qualitative (interviews & focus groups)	-	-	-	X	-	-	X	X	-
Andress & Fitch [61]	Six West Virginia counties, USA	Rural	*N* = 30 women, aged 21 years or older	Cross-sectional: qualitative (focus groups)	-	-	-	X	X	-	X	-	-
Bardenhagen et al. [62]	Michigan, USA	Rural	*N* = 20 food stores (7 small grocers/convenience stores without gas, 6 small or mid-sized grocers with gas, 5 mid-sized independent grocers, & 2 limited assortment/food mart/gas stations) *N* = 10 store owners, food bank representatives & local stakeholder	Cross-sectional: mixed methods	X	X	-	X	X	-	X	-	-
Breyer & Voss-Andrea [63]	Portland Oregon, USA	Urban	*N* = 204 stores (79 grocery stores, 51 chain stores & 74 other stores)	Cross-sectional	X	X	-	-	X	X	-	X	-
Bridle-Fitzpatrick [64]	Mazatlán, Sinaloa, Mexico	Urban	*N* = 20 mothers *N* = 593 food stores	Cross-sectional: mixed methods	X	X	-	X	X	X	-	-	-
Burns & Inglis [65]	Melbourne, Australia	Urban & Semi-rural	*N* = 15 supermarkets *N* = 33 FF restaurants	Cross-sectional	X	-	-	-	-	-	X	-	-
Cassady et al. [66]	Sacramento & Los Angles, USA	Urban	*N* = 25 supermarkets	Cross-sectional	-	-	-	-	-	X	-	-	-
Chen et al. [67]	Franklin county, Ohio, USA	Urban	*N* = 284 green retailers	Cross-sectional	X	-	-	-	-	-	X	-	-
Childs & Lewis [68]	Cherry Hill, Baltimore, USA	Urban	*N* = 15 supermarkets *N* = 33 FF chain outlets *N* = 30 community members	Cross-sectional: mixed methods	-	X	-	X	-	-	X	-	-
de Menezes et al. [69]	Belo Horizonte, Brazil	Urban	*N* = 336 food stores *N* = 2944 adults, aged 20 years & older, mean age 56.8 years, 88.4% female	Cross-sectional: mixed methods	-	X	X	X	X	-	X	-	-
Diehl et al.[70]	Denver, Colorado, USA	Urban	*N* = 5 neighborhoods *N* = 69 food stores (10 grocery stores, 27 convenience stores, 11 deep-discount stores, 5 neighborhood markets & 16 neighborhood markets catering to specific ethnic groups) *N* = 926 participants	Cross-sectional: mixed methods	X	X	-	X	X	-	-	X	-
Diez et al. [71]	Los Rosales, Madrid, Spain	Urban	*N* = 114 food stores & one street market *N* = 12 adults (6 men & 6 women), mean age 58.7 years)	Cross-sectional: mixed methods	X	X	-	X	X	-	X	X	-
Diez et al. [72]	Villaverde, Madrid, Spain	Urban	*N* = 24 residents, mean age 51.4 years	Cross-sectional: qualitative (participatory)	-	-	-	X	-	-	X	-	-
Duran et al. [73]	Sao Paulo, Brazil	Urban	*N* = 1842 adults aged 20—59 years, mean age 36.5 years, 53% female	Cross-sectional	X	X	X	-	X	X	X	-	X
Flint et al. [74]	Philadelphia, USA	Urban	*N* = 1263 adults, mean age 48 years *N* = 2 neighborhoods	Cross-sectional	-	X	X	-	X	-	-	X	X
Gao et al.[75]	New York, Baltimore city and county, Forsyth County, St Paul, Illinois and Los Angeles County, USA	Urban	*N* = 3634 adults, aged 45–84 years, mean age of 60.3 (SD 9.5), 51.3% female	Cohort	X	-	X	-	-	-	-	-	-
Glickman et al.[76]	Cleveland and Columbus USA	Urban	*N* = 449 adults (239 in Cleveland & 210 in Columbus) *N* = 2 neighborhoods considered food deserts	Cross-sectional	-	X	X	-	-	-	X	-	-
Gravina et al. [77]	Bilbao, Spain	Urban	*N* = 23 participants *N* = 3 neighborhoods	Cross-sectional: qualitative (participatory)	-	-	-	X	X	-	-	-	-
Harbers et al. [78]	Utrecht, Netherlands	Urban	*N* = 15 participants (5 males & 10 females) aged 33 – 79 years	Cross-sectional: qualitative (interviews)	-	-	-	X	-	-	-	-	-
Haynes-Maslow et al. [79]	North Carolina, USA	Urban	*N* = 68 low-income adults, 67.7% Black, 69.1% female	Cross-sectional: qualitative (focus groups)	-	-	-	X	-	-	X	-	-
Haynes-Maslow et al. [80]	12 community sites North Carolina counties, USA	Urban	*N* = 201 adults	Cross-sectional	X	-	-	-	-	-	X	-	X
Hendrickson et al. [81]	Minnesota, USA	Urban & Rural	*N* = 23 food stores *N* = 41 community residents, nutrition professionals & community leaders	Cross-sectional: mixed methods	X	X	-	X	-	-	X	-	-
Holston et al. [82]	Louisiana, USA	Rural	*N* = 44 adults (36 women & 8 men)	Cross-sectional: qualitative (focus groups)	-	-	-	X	-	-	X	-	-
Jiang et al.[83]	Massachusetts, Illinois, Iowa, USA	Urban	*N* = 142 adults (26 males & 116 females)], mean age 73.9 (SD 9.6) years	Cross-sectional: mixed methods	-	-	-	X	-	-	-	-	-
Jillcott et al.[84]	Pitt & Greene County, North Carolina, USA	Urban & Rural	*N* = 23 rural & urban women, aged 23—70 years	Cross-sectional: qualitative (interviews)	-	-	-	X	-	-	X	-	-
Karpyn et al. [85]	Philadelphia & Trenton, USA	Urban	*N* = 29 supermarkets *N* = 31 corner stores *N* = 796 adults (primary household food shoppers)	Cross-sectional	X	X	X	-	X	X	-	X	-
Ko et al. [86]	Washington State, USA	Rural	*N* = 57 food stores *N* = 69 restaurants *N* = 32 community residents, mean age 35.6 (SD 6.2) years	Cross-sectional: mixed methods	-	X	-	-	X	-	X	-	-
LeDoux & Vojnovic [87]	Detroit, Michigan, USA	Urban	*N* = 258 households	Cross-sectional	X	-	X	-	-	-	X	-	-
Leonard et al.[88]	Dallas Texas, USA	Urban	*N* = 298 neighborhood residents	Cross-sectional	-	-	X	-	-	-	X	-	-
Libman [89]	Brownsville & Upper East side, New York, USA	Urban	*N* = 22 residents & store workers (12 Brownsville & 10 Upper East side)	Cross-sectional: mixed methods	X	-	X	X	-	-	X	-	-
MacNell et al.[90]	North Carolina counties, USA	Urban & rural	*N* = 3 counties *N* = 42 women *N* = 28 food stores	Cross-sectional: mixed methods	-	X	-	X	X	X	-	-	-
Pessoa et al. [91]	Belo Horizonte, Brazil	Urban	*N* = 5611 adults, aged 18 years and older, mean age 39.7 years, 54.8% female	Cross-sectional	X	-	X	-	X	-	X	-	-
Rodriguez & Grahame [92]	Pennsylvania, USA	Urban	*N* = 11 adults (6 women & 5 men)	Ecological study: mixed methods	-	-	X	X	-	-	-	-	-
Rummo et al.[93]	Four US cities	Urban	*N* = 3299 adults	Cohort	X	-	X	-	X	-	-	-	-
Sharkey et al. [94]	Texas Brazos Valley, USA	Rural	*N* = 1409 adults	Cross-sectional	X	-	X	-	-	-	X	-	-
Tach & Amorim [95]	Philadelphia, USA	Urban	*N* = 66 adults *N* = 3 neighborhoods	Cross-sectional	-	-	-	X	-	-	X	-	-
Thornton et al. [51]	Melbourne, Australia	Urban	*N* = 1399 women *N* = 45 neighborhoods *N* = 134 food stores	Cross-sectional	-	X	X	-	X	X	-	-	-
Thornton et al. [96]	Melbourne, Australia	Urban	*N* = 932 women, mean age 33.3 (SD 7.6) years	Cross-sectional	X	-	X	-	-	-	X	-	-
Thornton et al. [97]	Victoria, Australia	Urban	*N* = 4335 women, mean age 34 years	Cross-sectional	X	-	X	-	-	-	X	-	-
Valdez et al. [98]	Merced County, South Merced and Winton, USA	Rural	*N* = 79 adults, mean age 41.6 years; 72% female; 79% Latino 53% Spanish-speaking	Cross-sectional: mixed methods	-	X	-	X	X	-	X	-	-
Vallianatos et al. [99]	3 Los Angeles Communities, USA	Urban	*N* = 1023 food outlets *N* = 10 community members	Cross-sectional: mixed methods	-	X	-	X	X	-	X	X	-
Walker et al. [100]	Pittsburgh, USA	Urban	*N* = 25 (men & women)	Cross-sectional: mixed methods	-	-	-	-	X	-	-	-	-
Wang & Qiu [101]	Edmonton, Canada	Urban	*N* = 96 supermarkets *N* = 47 local grocery stores *N* = 61 community gardens *N* = 17 farmers’ market *N* = 247 residential neighborhoods	Cross-sectional	X	-	-	-	-	-	X	-	-
Waters et al. [102]	Virginia & North Carolina, USA	Rural	*N* = 813 residents *N* = 483 food outlets (295 restaurants & 188 stores)	Cross-sectional	X	X	X	-	X	-	-	-	-
Williams et al. [103]	Australian community, Australia	Urban	*N* = 355 women, mean age 49.5 (SD 10.89) years	Cross-sectional	-	X	X	-	X	-	X	-	-
Zenk et al. [104]	Chicago IL, USA	Urban	*N* = 30 women, aged 21- 45 years	Cross- sectional: qualitative (interviews)	-	-	-	X	X	-	X	-	-
Zhao et al.[105]	Chicago IL, USA	Urban	*N* = 228 women, aged 18—44 years	Cross-sectional	-	X	-	-	-	X	X	-	-

*FF* Fast food

*GIS* Geographic information system

### Assessing associations between retail food environment exposures and food choices

Table 2 shows the studies that assessed the association between the local retail food environment and food choice using Geographic Information Systems (GIS)-based measures and store audits/surveys. Of the 19 studies that were included, six examined both the community and consumer food environment [51, 70, 73, 76, 85, 102], ten assessed only the community food environment [75, 80, 87, 88, 91, 93, 94, 96, 97, 103] and three assessed only the consumer food environment [69, 74, 105]. Local retail food environment exposures included availability (*n* = 8), accessibility (*n* = 13), perceived access (*n* = 2), healthy food availability (*n* = 8), perceived healthy food availability (*n* = 2), perceived consumer food environment (*n* = 1), perceived quality (n = 1), price (*n* = 6), quality (*n* = 6), variety (*n* = 2), in-store marketing (*n* = 6) and product placement (*n* = 6). Thirteen studies used GIS-based measures to describe the local retail food environment and geocode study participants’ homes and/or store types /outlets. The most used GIS-based measure was accessibility, which was measured as road network distances, Euclidean distances, straight line distance, travel times or spatial interaction models. The second most used GIS-based measure was availability which was measured as presence, ratio, variety, counts (within buffers) or relative density or probability density or kernel density of food stores. Some studies used GIS-based measures along with retrieving registered food store information using business directories and government databases. The use of GIS-based methods to analyze the availability and accessibility of food stores has been discussed in previous reviews [10, 11]. Only one study used global positioning system (GPS) to assess the community food environment [64].

**Table 2:** Studies assessing the association between the local retail food environment and food choice in studies using GIS-based measures and store audits/surveys

Author (Year)	Sample size (n)	Food Environment exposure (GIS Based and/or store audits)	Food choice outcome (Dietary intake)	Results
Diehl et al. [70] Mixed methods	*N* = 5 neighborhoods *N* = 69 food stores *N* = 926 participants classified into three social groups: advantaged, middle, and disadvantaged (low income Black and Hispanic females)	- Food stores were geocoded using ArcMap. Community variables assessed included distance and density - Consumer environment assessed using Healthy Food Basket (15 food items), measured affordability and availability	- Self-reported frequency consumption of FV, SSB, meats, FF & snacks	- Proximity to a grocery store was associated with lower consumption of FV, higher consumption of SSB, and lower consumption of healthy proteins (beans, chicken, and fish) - Higher density of grocery stores was associated with lower consumption FV, higher consumption of SSB, and higher consumption of unhealthy fats. Disadvantaged participants had more food stores and grocery stores within 1 mile - Affordability (price) was not associated with food intake. For the disadvantaged group, average cost of a Staple Food Basket was less expensive at the closest store, but more expensive at the closest grocery store and the preferred store respectively - Increased availability of healthy food items at the closest grocery store was associated with lower consumption of SSB - The disadvantaged group did not have a significantly greater number of available healthy food items at the closest food store or at the preferred store, but they did have fewer available healthy items at the closest grocery store
de Menezes et al. [69] Mixed methods	*N* = 2944 adults, aged 20 years & older, mean age 56.8 years *N* = 336 food stores	- Food and vegetable food store audits - Consumer nutrition environment variables were assessed using the ESAO-S - The ESAO-S healthy food access was summarized by the HFSI	- Questions adapted from international surveillance systems used to assess daily FV consumption	- No associations found between FV intake and local grocery stores food availability, variety, quality, pricing, signage, and promotion - Both HFSI and specialized FV markets were positively associated with F&V intake, but not with FV quality
Duran et al. [73]	*N* = 1842 adults aged 20—59 years, mean age 36.5 years	- Community food environment measures included density of and proximity to supermarkets and fresh produce markets - Consumer environment measures included availability, price, quality, and variety of fresh FV and SSB, assessed using the ESAO-S	- Questionnaire- consumption of FV and SSB (≥ 5 days/week)	- Lower income individuals living in neighborhoods with lower density of supermarkets and fresh produce markets had statistically significantly lower FV consumption - FV availability was associated with a 41% increase in the prevalence of regular FV consumption. FV price was not associated with FV consumption - Price, supermarkets and fresh produce markets density or proximity were not associated with SSB consumption
Flint et al. [74]	*N* = 1263 adults, mean age 48 years *N* = 2 neighborhoods Both sites had two grocery stores & 55/56 convenience stores	- Consumer food environment assessed using NEMS	- Block FFQ—measured portions of FV consumed per day	- Consumer food environment measures (availability, price, quality, and marketing) was not associated with and FV consumption, neither in bivariate nor multivariate analyses - Participants who perceived their neighborhood to have more variety and higher quality grocery stores did not have a higher daily FV intake compared to those perceived their neighborhood to have little choice and low quality grocery stores - No difference in intake between participants who perceived neighborhood to have higher choice and higher quality of food available and those who perceived neighborhood to have low quality and little choice
Gao et al. [75]	*N* = 3634 adults, aged 45–84 years, mean age 60.3 (SD 9.5), 51.3% females	- GIS measures: 1. GIS-derived distance to the nearest favorable food store, 2. GIS-derived one- mile kernel density of favorable food stores, 3. survey-based measure of perception of healthy food availability, and 4. summary measure combining GIS-derived one-mile kernel density of favorable food stores and survey-based measure of participants’ perception of healthy food availability	- Diet assessed using AHEI Index score	- Higher AHEI score was associated with shorter distance to nearest favorable stores, higher SES neighborhood, better perception of healthy food access, and higher composite score of healthy food environment
Glickman et al. [76]	*N* = 449 adults	- Food retail audits- data collected on availability, price and quality of healthful foods - PFRQ score calculated using audit adapted NEM-CS and BTG-COMP	- Three 24-h dietary recalls: Average HEI-2010 score, average SSB intake, and average FV intake	- No associations between PFRQ and HEI-2010 scores for participants who shop further from home - An increase of one unit in PFRQ score was associated with a 14.7-point increase in HEI-2010 score for residents who shopped close to home - No association between PFRQ and FV and SSB consumption. Higher quality proximate food retail was associated with improvements in diet
Haynes-Maslow et al. [80]	*N* = 201 adults	- Self-reported FV access was measured using three neighborhood perceived access questions adapted from previous studies - Food outlet density (within 1 mile of participants home)	- FV intake (cups per day) was assessed using the 10-item National Cancer Institute FV screener	- Positive correlation between perceived access to FV variety and of supermarkets within 1 mile of a participant’s home - Positive association between perception based FV access measures and objective measures (food outlet density within 1 mile of participants home) - No association between perception-based measures (convenience, variety, and quality) and FV intake - Association between access to supercenters within a mile of participants' houses and lower self-reported intake of FV - Participant's FV intake decreased by 0.61 cups per day when there were more supermarkets within a mile of their home
Karpyn et al. [85]	*N* = 29 supermarkets *N* = 31 corner stores *N* = 796 adults	- GIS based measures- supermarkets within a two-mile buffer corner stores within both three-square-mile study areas - Nutrition environments in supermarkets and cornerstores using NEMS-S and NEMS-CS tools. Both types of retail outlets were scored on availability, price, and quality of both healthier and less-healthy food items	- 24-h dietary recall: HEI score and fruit consumption subscore, and vegetable consumption subscore)	- Store quality and perceived neighborhood food availability was positively related to vegetable consumption sub scores
Leonard et al. [88]	*N* = 298 neighborhood residents	- Access to food sources calculated in ArcMap using straight-line distances between the respondent’s address and the location of food sources	- Neighborhood (Perceived Good Access) and the number of meals prepared at home in a typical week	- Residents living closer to fresh food sources consumed more FV, while those living closer to FF restaurants consumed less FV
LeDoux & Vojnovic [87]	*N* = 258 households	- ESRI Network Analyst in ArcMap 10.1 used to measure accessibility. Distance from resident’s house to nearest store category	- Respondents were asked to recall separately their typical daily, weekly, and monthly servings of soda, fruit juice, sweets, salty snacks, FV	- Closer proximity (quarter mile = 402 m) to a supermarket was associated with increased intake of both healthy and unhealthy food groups - Short and medium (half mile) proximity to FF outlets was associated with lower FV consumption - Lower FV consumption when there are more FF outlets within a quarter and half-mile (402 and 805 m), respectively
Pessoa et al. [91]	*N* = 5611 adults, aged 18 years and older, mean age 39.7 years	- Density of supermarkets and hypermarkets, density of mini markets, grocery stores and warehouses, density of healthy food outlets (stores and open-air markets specialized in selling FV), density of restaurants and density of unhealthy food outlets (bars, snack bar and food trucks/trailers)	- Questions were used to estimate the daily frequency of fruit intake. FV intake score	- High income neighborhood and higher density of healthy food outlets associated with higher FV intake scores - High density of unhealthy food outlets was associated with lower FV intake score
Rummo et al. [93]	*N* = 3299 adults	- GIS used to capture all food outlets within a 5-mile radius of each participant - Availability of food stores: calculated the count of each type of food resource within a 3 km distance along the street network around participant homes	- Questionnaire used to calculate Priori diet quality score - Food groups: fruits vegetables, whole grains, processed meats, snacks, desserts, SSBs and Artificially sweetened beverages (ASBs)	- For participants with lower individual-level income, the availability of neighborhood convenience stores was associated with lower diet quality - The percentage of neighborhood convenience stores relative to total food stores and restaurants was negatively associated with whole grain consumption; these associations were stronger at lower (vs higher) individual-level income - No associations between FV and processed meat consumption and community nutrition measures - Consumption of SSBs and ASBs and number of SSBs and ASBs consumed were not associated with neighborhood convenience stores' availability
Sharkey et al**. **[94]	*N* = 1409 adults	- Two measures of potential spatial access: proximity (distance to the nearest location) and coverage (number of traditional FF restaurants, non-traditional FF outlets, and all FF opportunities from each respondent’s residence within 1, 3 or 5 miles). All participants were geocoded to their residence	- Questionnaire—Weekly consumption of FF meals	- FF meals were consumed less frequently when proximity from a FF restaurant, non-traditional FF outlet, or all FF outlets was greater - Closer distance and greater coverage were associated with more frequent consumption of FF meals by women than men
Thornton et al. [51]	*N* = 1399 women *N* = 45 neighborhoods *N* = 134 food stores	- Community nutrition environment: locations of greengrocers, major supermarkets, and FF restaurants in and immediately surrounding the neighborhoods - GIS was used for geocoding of participants and food stores. Proximity- distance between each participant’s household location and the nearest store of each type (greengrocer, supermarket, FF restaurant). Density-count of each store type within 3 km of road network distance from each participant’s household. Opening hours measures were calculated for each store type - Consumer nutrition environment: store audits on the availability and price of 15 commonly consumed fruits and 23 vegetables in 134 stores, identified as being within the boundaries of the 45 neighborhoods	- Questionnaire—FV and FF consumption	- FV prices were lower in greengrocers in highly disadvantaged neighborhoods, but operating hours and availability were more restricted compared to other neighborhoods - Residents in high-disadvantaged neighborhoods were more likely to live further from a FF restaurant and have lower density and variety of chains than those in low-disadvantaged neighborhoods - Fruit consumption was not associated with neighborhood-level disadvantage - Participants in high disadvantaged neighborhoods were significantly less likely to consume two or more servings of vegetables per day - A greater density of greengrocers and supermarkets in the neighbourhoods of frequent vegetable consumers, as well as a greater variety of vegetables in greengrocers, were factors contributing to their frequent consumption of vegetables - Prices in both greengrocers and supermarkets were positively associated with consumption of FV
Thornton et al. [96]	*N* = 932 women, mean age 33.3 (SD 7.6) years	- GIS—count of FF restaurants within a 3 km road distance	- Two questions on FF consumption	- Women with moderate or low confidence in shopping for healthy food had significantly lower odds of rarely consuming FF in comparison with women with the highest confidence - Women who live more than 1.6 km from their nearest supermarket are significantly less likely to consume FF infrequently compared to women living within 0.8 km from their nearest supermarket
Thornton et al. [97]	*N* = 4335 women, mean age 34 years	- Geocoding household addresses of participants. ArcGIS 9. 3, used to identify and calculate the number of chain supermarkets and greengrocers within a 2 km road network distance from each individual’s household location	- Self-reported FV consumption (servings per day)	- Store access within 2 km may moderate the association between vegetable consumption and cooking confidence, though evidence is weak (*P* = 0.062)
Waters et al. [102]	*N* = 813 residents *N* = 483 food outlets (295 restaurants & 188 stores)	- Availability of healthy food in food outlets was assessed by the NEMS-S and NEMS-R. Data from store audits used to calculate a healthy food availability score - All food outlets were geocoded and mapped in ArcGIS 10.0 to determine proximal food environment to food outlets	- Self-reported FV intake (cups/day) was assessed using the National Cancer Institute’s FV short screener	-No association between FV intake and healthy food availability in food outlets
Williams et al. [103]	*N* = 355 women, mean age 49.5 (SD 10.89) years	- Supermarkets and FV store’s locations and participants addresses were geocoded in the GIS ArcView 3.3	- Two questions on FV consumption (servings/day)	- High fruit consumers were mainly women of older age, dieting to lose weight, preferred fruit, and perceived that more healthy food options were available, and that fruit cost less
Zhao et al. [105]	*N* = 228 women, aged 18—44 years	- Consumer food environment aspects price, availability, marketing, and product placement assessed using an instrument combining the NEMS and the Bridging the Gap Food Store Observation Form, audits. The NEMS-P was used to assess participants’ perceptions of the consumer food environment	- Self-reported FV consumption (cups/day) using 6 item FFQ	- In the multivariable regression analyses no significant association between any measure of the consumer food environment (price, availability, marketing, and product placement) and fruit intake was found - Vegetable intake was associated only with marketing in the consumer food environment. Greater Healthy food marketing exposure was associated with approximately 0.24 cups increase in vegetables consumed per day by participants

*ASB* Artificially sweetened beverages (fruit drinks, soft drinks, and water)

*AHEI* Alternative healthy eating index

*BTG-COMP* Bridging the gap community obesity measures

*ESAO-S*s Obesogenic environment study food store observation tool

*FFQ* Food frequency questionnaire

*FV* Fruits and vegetables

*GIS* Geographic information system

*HEI* Healthy eating index

*HFSI* Healthy food store index

*NEMS* Nutrition environment measures survey

*NEMS-C* Nutrition environment measures survey corner store

*NEMS-P* Perceived nutrition environment survey

*NEMS-R* Nutrition environment measures survey restaurant

*SEP* Socioeconomic position

*SES* Socioeconomic status

*SSB* Sugar-sweetened beverages

A variety of stores were included in most of the studies. The most common store types were grocery stores, supermarkets, convenience stores, FF restaurants, green grocers, and farmers markets. Tools to measure the consumer food environment were the Nutrition Environment Measure Survey (NEMS) (*n* = 4), Obesogenic Environment Study food store observation tool (ESAO-S) (*n* = 2), Bridging the Gap Community Obesity Measures project (*n* = 2), healthy food basket (*n* = 1) and store audit (*n* = 1). Only one study used the NEMS-R to collect information on restaurants, and one used the NEMS-P to assess perceptions of the consumer food environment. Food choices/dietary outcomes examined included FV intake (*n* = 15), FF consumption (*n* = 4), SSB intake (*n* = 4), snacks (*n* = 2), food groups (*n* = 2) and dietary quality indices such as Healthy Eating Index (HEI) (*n* = 2), Alternative Healthy Eating Index score (AHEI) (*n* = 1) and A Priori diet quality score (*n* = 1). Most studies (*n* = 17) used questionnaires (set questions or food frequency questionnaires) to assess food choices and two studies assessed dietary intake using 24-h recalls (*n* = 2).

### Community food environment and consumption of healthy and unhealthy food*s*

Four studies found no association between proximity to grocery stores or supermarkets and FV intake [69, 70, 76, 80], and one study found no association between accessibility to supermarkets or green grocers and vegetable consumption [75]. Living near a fresh food source was associated with higher FV consumption [70]. A greater density of greengrocers and supermarkets was associated with frequent consumption of vegetables[51]. Living close to a FF restaurant [87, 88], and a higher density of grocery stores [70], supercenters and supermarkets [80] and unhealthy food stores such as bars, snack bars and food trucks within neighborhoods were associated with lower FV intake [91].

A cross-sectional study in the USA found an association between closer proximity to a supermarket and higher intake of both healthy and unhealthy food groups respectively [87]. Another study in the USA reported no association between living in closer proximity to grocery store and consumption of healthy proteins like beans, chicken and fish, but higher density of grocery stores was associated with eating unhealthy fats [70].

With regards to SSB, one study in the USA reported that closer proximity to and higher density of grocery stores were associated with greater consumption of SSB [70], while another USA study showed no association between proximity to healthy food stores and SSB consumption [76]. Although availability of convenience stores was associated with lower diet quality in low-income individuals in four USA cities it was not associated with SSB consumption [93]. Also, a Brazilian study reported that proximity to and density of supermarkets and fresh produce were not associated with SSB consumption [73].

Five studies assessed the association between community food environment and FF consumption. Living further away from a FF restaurant (including traditional, non-traditional or all FF) [94] or a healthy food source such as a supermarket [96] was associated with lower FF consumption. Highly disadvantaged neighborhoods in comparison to low disadvantaged neighborhoods had lower density and variety of FF restaurants [51].

### Community food environment and overall diet quality

Closer proximity to healthy food stores was associated with higher HEI scores [76], and closer proximity to supermarkets was associated with higher AHEI scores [75].

### Consumer food environment and consumption of healthy and unhealthy foods

A Brazilian study found no relationship between grocery stores and FV intake however, better access to healthy foods in stores and specialized FV markets was associated with greater FV intake [69]. In contrast, a study in rural USA found no association between healthy food availability and FV intake [102]. In another USA study, perceived neighborhood food availability was associated with higher vegetable consumption [85]. An Australian study reported that higher perception of healthy food availability and perceived lower cost of fruit was associated with high fruit consumption [103]. A USA study reported a negative association between availability of healthy food in stores and SSB consumption [70]. An Australian study reported that prices in both greengrocers and supermarkets were positively associated with consumption of FV[51]. Affordability (price) was reported not to be associated with overall food intake [70] and FV and SSB consumption [73], while marketing was positively associated with vegetable consumption [105]. Perceived greater variety of stores and quality of local grocery stores was not associated with consumption of FV [74].

### Barriers and facilitators for access to healthy food in resource-poor communities

#### Qualitative studies

Table 3 shows the barriers and facilitators for access to healthy food in resource-poor communities as reported in nine qualitative and eleven mixed method studies. In resource-poor communities, high food costs were cited as the main barrier to healthy food access [60, 62, 71, 78, 79, 81, 82, 86, 92, 95, 98, 104]. The second major barrier to healthy food access was transportation (lack of public transportation or car ownership) [61, 62, 77, 79, 81, 82, 84, 90, 92, 95]. Seven studies reported geographic access as barrier to healthy food access [61, 71, 81, 84, 89, 92, 95]. Five studies reported the presence of unhealthy food stores such as corner /convenience stores and FF restaurants as barrier to healthy food access [77, 79, 82, 89, 95]. A lack of healthy food availability [60, 104], the presence of unhealthy foods in various stores [71, 77] and lack of quality and variety FV [79, 81, 104] were perceived as barriers to healthy food access in the consumer food environment. Two studies reported that living in a food desert was a barrier to healthy food access [100, 104].

**Table 3:** Barriers and facilitators for healthy food access in resource poor communities identified in qualitative and mixed methods

**Author (Year)**	**Themes**	**Barriers & Supporting quote or data**	**Facilitators** &** Supporting quote or data**
Alkon et al. [60]	1. How do people think about food access?	- Neighborhood’s underdevelopment - Lack of control over the food stores in neighborhoods - Lack of fresh food in their neighborhoods - Price is the primary barrier to food access	N/A
Andress & Fitch [61]	1. Structure of place, external food environment	- Geography of place created barriers getting to and from grocery stores and other sources of food - Lack of car ownership and no public transportation	N/A
Bardenhagen et al. [62] Mixed methods	1. Transportation challenges 2. Cost of healthy eating	- Distance and cost are a large barrier to accessing healthy foods - Transportation is one of the largest barriers to accessing healthy food - Higher cost of healthy foods - High food prices may hinder the use of farmers markets “*Everything is more expensive here. It’s very much a third-world mentality*.”	- Interest in locally grown food “*More and more people in the region want to know where their food supply is coming from, but low-income people can’t always pay the price*”
Diez et al**. **[71] Mixed methods	1. Community food environment 2. Consumer food environment	Community food environment - Accessibility-related aspects (‘poor access built environment obstacles’) - Unhealthy foods within food stores, bars and restaurants perceived to negatively influence diets Consumer food environment - Unhealthy food presence - Cost barriers *“It’s the same thing with the organic food shops, they sell very healthy products, but they are quite expensive … quite expensive. Very healthy, but not affordable.”*	Community food environment - Small, specialized stores offered a wide variety of healthy foods **“***Neighborhood food stores have lots of fruits and vegetables*.” Consumer food environment - Availability of organic and dietetic food products
Diez et al. [72]	1. Food stores	N/A	- Small traditional food stores *“We have to protect these stores against other retail types such as supermarkets or street markets, especially in terms of places where you can by fresh food.****”*** - Presence of street markets offering a wide variety of affordable fresh foods
Gravina et al. [77]	1. Unhealthy eating behaviors 2. Retail transformation 3. Healthy eating	Unhealthy eating behaviors - Presence and affordability of FF and sugary food *“Fast food is not hygienic and healthy, but it is often cheaper and easier to get or consume.”* Retail transformation - Greater amount of convenience stores	Healthy eating - High quality of the foods offered by merchants in neighborhood
Haynes-Maslow et al. [79]	1. Community-level barriers	- Cost barrier to purchasing fresh FV *“What we need to eat — and what we want to eat — the price is a big part of it. When you have lower-income families, they usually don’t introduce fruit and vegetables into their children’s body because it costs so much. So, if there were... if there was a price where everybody could afford it, then everybody could have it.”* - Transport was a barrier to purchasing FV, especially for the elderly or those who did not own a vehicle - Lack of quality FV in the community *“I tell them, “Look, these apples are rotten.” They’re pretty on the outside, but they’re rotten. So, they gave me another one... that was rotten, as well.”* - Lack of variety of FV in grocery stores in the community *“You can get vegetables but not the variety of vegetables that you might want.”* - Changing food environment – Farm markets and roadside stands declined, and FF restaurants in the community increased *“He had an old truck and he sold vegetables out of his truck... for me, I don’t see him no more... but that would be nice if we had somebody who would come around with vegetables.”*	N/A
Harbers et al**. **[78]	1. Environmental Determinants of Food Choice	- Increasing prices of fresh FV *“But if I see that snacks are cheap, and that fresh food is only getting more expensive... And if you only receive social assistance benefits. That is just undoable.”* - Marketing strategies and food product placement in the supermarket perceived as tempting and encouraged unhealthy food purchases *“They should put this on more products. So, if you are in front of the crisps shelf, you can think, well, I can take Lays because that is easy. But that then you have an alternative next to it, from which you can see, well, it does actually provide less calories and it is just as tasty.”*	N/A
Hendrickson et al. [81] Mixed methods	N/A	- Lack of transportation - Lower quality and higher food prices in urban community - Distance and cost to go to stores for rural residents Rural residents, 85–90% drive to food stores Urban residents, 40–60% walk and 15–30% take the bus - Healthy food choices not affordable within communities and participants believed that people in their community were food insecure	N/A
Holston et al**. **[82] Mixed methods	1. Store Choice: 1.1 Outshopping Convenience Stores/Dollar Stores	- Participants perceived cost as a barrier to providing healthy foods for their families Convenience Stores/Dollar Stores - No participants viewed convenience stores as a viable option for acquiring food	- Outshopping- having to leave the parish to find lower prices and better quality - Ways of Acquiring Food All focus groups reported using a variety of built, cultivated, and wild environments to acquire food - Facilitators other than the grocery store - Gardening - Fishing and hunting
Jiang et al. [83] Mixed methods	N/A	N/A	- The key factors to contributing to consumption of FV were accessibility and affordability, while living accommodations were least important factor - Lower FV prices In addition to lowering FV prices, participants suggested stores have more sales or discounts on FV and donate fruit and vegetables to seniors. Participants suggested that food stores should *“have a clearance section,” “lower prices or (have) more sales on fruits and vegetables,”* and *“sell fruits and vegetables that are less satisfying in quality cheaper.”* - Sales and discounts on FV - In store marketing strategies Participants recommended stores improve the presentation and placement of FV. Participants suggested that food stores “keep them [fruit and vegetables] close to the door” and “*display them nicely so [customers] want to buy them.” “Farmer’s markets should open for longer time through the season.”*
Jillcott et al. [84]	1. Farmers market 2. Supermarkets 3. Discount supercenters	- Farmers market is far away from residence and transportation is needed to access them. Fresh food markets need to be closer to residence	- Supermarkets-high quality meats, lower food costs, convenient locations - Discount supercenters – offered lower food prices
Ko et al. [86] Mixed methods	1. Perceived accessibility of fresh produce 2. Food store preference	- Perceived accessibility of fresh produce accessibility was dependent on farming seasons which impacts seasonal prices	- Large grocery stores chosen based on food quality, price, and nearest location to their home
Libman [89] Mixed methods	N/A	- Lack of food availability in the neighborhood, means residents have to go outside neighborhood to get affordable healthy foods - Greater presence of convenience stores & FF in low-income communities compared to higher income communities	N/A
MacNell et al. [90] Mixed methods	1. Food prices 2. Freshness and variety 3. Access to transportation	- Lack of transportation	- Lower prices - Freshness and variety
Rodriguez & Grahame [92] Mixed methods	1. Cost 2. Transportation 3. Access to services 4. Education and information	- Cost: primary factor in food choice. Proximity to stores also complicated the cost of food, more affordable stores were further away from the community - Transportation: lack of public transportation or personal vehicle, difficult terrain to walk, physical disability and transportation costs - Access to services: Lack of access to services such as WIC, food pantry or DPW affects food access - Education and information: no internet access meant inability to retrieve money-saving coupons, recipes, or information regarding health concerns. Lack of information available about where they could use food stamps or access fresh food	N/A
Tach & Amorim [95]	1. Grocery Shopping: Choice within Constraint 2. Alternative Food Acquisition Strategies	- Economic Constraint: High food cost - Geographic Constraint: Distance affected accessible stores especially for residents without cars - Alternative Food Acquisition Strategies: Corner stores with high prices and low quality foods. No presence of farmers markets - Opening of high-quality stores in neighborhoods – participants perceived these as overpriced	- Economic Constraint: Participant using strategies such as buying from multiple stores, buying in bulk, or buying store brands - Geographic Constraint: Car owners can travel further to stores that are cheaper or offer higher-quality products - Charitable Donations: charitable food sources including nonprofit food pantries and programs and food distribution events at nearby churches, schools, and public housing projects
Valdez et al. [98] Mixed methods	1. Access to FV 2. Affordability of FV	- Affordability of FV: most (65%) reported that “healthy food options like FV are too expensive in retail stores *“It would be nice to be able to have a store that you can actually afford to go to...even the dollar store isn’t the dollar store anymore.”*	- Living in an agricultural setting with access to farmers and workers and mobile fruit vendors Survey respondents agreed with the statement *“A large selection of fruits and vegetables is available in my neighborhood”* and *“The fruits and vegetables in my neighborhood are of high quality.” “You can buy vegetables all over the place.”* - FV stands and flea markets, as opposed to retail stores, were good sources for cheap produce
Walker et al. [100] Mixed methods	N/A	- Neighborhood store closure in food oasis *“The bad economy leads to poor neighborhoods and store closings. Poor neighborhoods end up losing the stores and now we need more neighborhood stores.”* - Food desserts: In food desserts, food was perceived as necessary for survival however macro-level factors influencing food purchasing *“Eating junk food is what I can afford.”* *“Corporate taking advantage of the consumer (by offering smaller food quantities for more money)”*	- Food oasis participants had access to organic foods, resources such as Catholic Charity, Salvation Army, senior coupons for farmer’s market, SNAP and WIC vouchers *“Organic food stores have decent prices and good quality.”* *“Shopping frequently for fresh produce.”*
Zenk et al. [104]	1. Material 2. Economic	- Material barriers: lack of a full-service supermarket in the neighborhood. Lack of store maintenance was associated with poor quality food. Neighborhood stores had low stock and variety of some foods (including fresh produce) and foods were of a poor quality. According to women, stores had expired canned and packaged foods, wilted fresh FV, and moldy spoiled meats - Economic barriers: high food prices at both small local stores and supermarkets	N/A

*FV* Fruits and vegetables

*FF* Fast food

*SNAP* Supplemental nutrition assistance program

*WIC* Women, infants, and children

In terms of store type, supermarkets, discount stores, large grocery stores and traditional stores, farmers markets and street vendors/ FV stands were perceived as major facilitators for healthy food access in resource poor communities [71, 72, 84, 86, 98, 100]. Three studies reported that lower food cost in food stores such as supermarkets, discount stores was a facilitator for healthy food access in resource poor communities [71, 82, 86]. Consumer food environment characteristics such as in-store availability of healthy foods [71], quality [77], marketing and sales [83] and variety [90] were also perceived as facilitators for healthy food access.

Two studies in the USA reported that food assistance from non-profit organizations and government programs such as the Supplemental Nutrition Assistance Program (SNAP) and Women, Infants, and Children (WIC) increased healthy food access for residents in communities [95, 100]. Individual strategies such as gardening, fishing and hunting [82], purchasing from various sources, buying in bulk and buying store brands [95] also enabled healthy food access.

#### Quantitative studies

Table 4 shows the barriers and facilitators for healthy food access in resource poor communities as reported in five quantitative and three mixed method studies. Living further away from grocery stores [63, 68, 99] and shorter operating hours of healthy food stores [67] were associated with poor healthy food access. Barriers to healthy food access include in-store high food prices [63, 99], unavailability of healthy foods [68] and product placement and promotion of unhealthy food items [64]. Access to healthy food was also limited by a lack of access to a car or lack of transportation [65] as well as neighborhood crime and safety issues [68, 99]. Facilitators for healthy food access include public markets [64], vehicle ownership [65], in-store prices [66], access to fresh produce and public transportation [101].

**Table 4:** Barriers and facilitators for healthy food access in resource poor communities identified in quantitative studies

**Author (Year)**	**Food Environment exposure** **(GIS Based and/or store audits)**	**Results**	**Barriers** &** Supporting quote**	**Facilitators & Supporting quote**
Breyer & Voss-Andrea [63]	- Healthful foods market basket survey biased on USDA Thrifty plan - Affordability index	- Store distance increases nonlinearly as affordability index declines, showing a negative correlation between affordability index and proximity to the nearest affordable store - On average, 1.6 miles (2.9 km) travel distance between a low-cost store and nearest grocery store; 65% of participants lived in either extreme or moderate food mirages	- High food prices - Proximity	N/A
Bridle-Fitzpatrick [64] Mixed methods	- Global positioning system (GPS) used to map food outlets in neighborhoods - Price index	- More unhealthy food stores in low- and middle-income communities compared to the high- income community - Greater exposure and access to fresh FV, SSBs and obesogenic snacks in low- and middle-income communities compared to the high-income community - Lowest-income community had the second highest prices for FV, eggs and dairy, and grains and other basic staples - Packaged snacks and SSBs prices were lowest in the lowest-income community - Photos used by participants to describe the food environment showed a high presence of SSBs and packaged snacks in their communities. Participants also stated that these unhealthy foods drew their attention	- High food prices - Displays and promotions for packaged snacks and soft drinks The racks of sweet and salty snacks *“called my attention”* or *“were most visible.”* The store “has almost nothing of healthy food.”	- Public market Regarding the public market *“because it is* *cheaper…. You can get a little bit of different vegetables for a low price.”*
Burns & Inglis [65]	- GIS modelling used to measure access to supermarket and FF and transport networks	-—More advantaged areas had closer access to supermarkets, less advantaged areas had closer access to FF outlets	- No car access	- Car access
Cassady et al. [66]	- Thrifty food plan market basket - 2005 Dietary guidelines market basket	- Both cities had significantly lower average total prices for thrifty food plan baskets of FV compared to retailers in middle- and high-income neighborhoods - Stores located in very low income neighborhoods and low income neighborhoods had similar FV prices. Low-income households would have to allocate 70% of their food budget to FV to meet the 2005 dietary guidelines	N/A	- Price
Chen et al.[67]	1. Spatial food access 2. Temporal food access 3. Spatiotemporal food access	- Lower SES neighborhoods have better spatial access but poorer temporal access than higher SES neighborhoods - Disadvantaged tracts have slightly more spatiotemporal advantage in food access	- Limited temporal access i.e., shorter healthful store operating hours	N/A
Childs & Lewis [68] Mixed methods	- Number of food stores available within an eight kilometer radius of neighborhood and store survey	- The least readily available food type in the survey was fresh fruit and vegetable - The most readily available foods were processed and canned foods: protein; non-dessert dairy; canned/frozen vegetables; grains and bread; and canned/frozen fruits - In Cherry Hill, 73% of respondents perceived FF to be more readily available than fresh foods - 50% of respondents said that a grocery store in the neighborhood would improve food access in the community - Travel time to the supermarket for most participants was 6–15 min; for some it was 30 min	- Lack of financial resources - Lack of stores carrying nutritionally appropriate food - Limited mobility due to the physical and built environment-long distance to travel to stores - Neighborhood crime - Time of individuals	Vehicle ownership *“When grocery stores open here, they are overpriced and never stocked with anything of value.”* *“They could improve in having meat, fruits and vegetables that aren’t spoiled.”* *“To open up a store that will be well stocked with quality food, healthy food and value conscious.”*
Vallianatos et al. [99] Mixed methods	- GIS mapping number the type of retail food outlets - Thrifty food plan- assessing availability and affordability of healthful foods	N/A	- Food is perceived to be expensive - Far distance to nearest supermarket means residence spend a lot of money on transport and gasoline (for car owners) and this causes reliance on convenience stores or other small stores near their homes - Violent crime makes it difficult for residents to shop after dark - FF can be easily purchased in the neighborhoods and outside school grounds	N/A
Wang & Qiu [101]	N/A	- Negative relationship between private vehicle access and number of food stores - Neighborhoods with a higher unemployment rate and residents who walk primarily to stores had a 0.42 and 0.14 likelihood of more super-markets and local grocery stores, respectively,	N/A	- Disadvantaged groups had higher access to fresh food sources - Public transport use

*FV* Fruits and vegetables

*FF* Fast food

*GIS* Geographic information system

## Discussion

This scoping review provides an overview of the evidence on adult food choices in association with the local retail food environment and barriers and facilitators for food access in resource-poor communities. Literature shows that food environments may differ across communities, neighborhoods, cities and countries [34]. In contrast to previous reviews that focused on the food environment in different countries, this review focused on studies that reported on low-income communities/neighborhoods and/or low-income households. Results on associations between food choice (dietary outcomes) and the local retail food environment were inconsistent. Numerous studies have stated that heterogeneity of measurement tools for the community and consumer food environment contribute to difficulty with interpreting study outcomes [8, 29, 32, 40, 43]. The standardization of measures to assess the food environment is therefore needed. Recent systematic reviews on food environment and diet in various settings also reported inconclusive findings [10, 35]. Similarly, also to other reviews, mostly cross-sectional studies were included and only two longitudinal studies were included in the present review. This scoping review shows that in resource-poor communities, cost, transportation, limited geographic access, and the presence of unhealthy food stores are the main barriers for access to healthy food. Facilitators that enable access to healthy food include store types such as supermarkets, large grocery stores and farmers markets, lower in-store food prices, food assistance programs, access to transportation, in-store availability, quality, and marketing of healthy food.

Many studies included in this review measured accessibility and availability of food stores within neighborhoods, and consumption of FV and SSB respectively were the most frequently studied dietary outcome. Other reviews have also reported that FV intake was the most common outcome measure [10, 28]. It has been postulated that accessibility to FV stores may influence consumption of FV [29]. In the present review, there was no association found between accessibility and FV intake, while retail food environments were associated with SSB consumption. This review has found little evidence to suggest that in resource-poor communities lower FF consumption is associated with inaccessibility and lack of FF restaurants. These findings suggest that greater access to FF restaurants may encourage unhealthy food choices that are contrary to dietary recommendations that aim to promote healthier food choices [27]. A few studies in the present review reported findings on the association between affordability, price, variety, marketing, quality, and placement (shelf space for healthier food products and unhealthy snacks and drinks), perceived consumer environment and food choices. No studies included in the scoping review reported on the association between food promotion (signage, in-store advertising, health/education materials near food products) and food choices.

In this scoping review, cost and transportation were identified as the two major barriers for access to healthy food in resource-poor communities. It is well known that cost is a barrier to healthy diets worldwide [106]. The availability of transportation allows residents to shop anywhere they can access healthy foods, even if these foods aren't readily available in their neighborhood [107]. This scoping review further shows that lower food prices and store types such as supermarkets, discount stores, large grocery stores and traditional stores, farmers markets and street vendors/ FV stands were considered major facilitators to healthy food access. Food pricing policies such as taxes, price manipulations of SSB, energy dense, low nutrient or high in added sugars or saturated fats and food subsidies on FV can promote healthy diets [106]. A systematic review reported that pricing interventions used in high- and middle-income countries positively affect consumer behavior and improve purchasing and consumption of healthy foods and beverages [108]. Another systematic review found, however, that while policies and FV subsidies are being implemented and supermarkets are becoming more common among resource-poor communities in an attempt to change diets positively [109], supply and demand issues have prevented the expected change [110]. Therefore, increasing proximity does not necessarily result in consumers purchasing and consuming more healthy foods. Sawyer and colleagues stated that for change in unhealthy food environments, creative strategies that support household finances at individual level and transform societal behavior to encourage healthy food production, supply and intake are needed [34].

In this scoping review, convenience/corner stores were also identified as a barrier to healthy food access in resource poor communities. Also, higher neighborhood density of convenience stores was shown to be associated with poor quality diets [93]. To encourage healthier food choices, stores can implement various in-store marketing, placement and pricing strategies as reported in studies conducted in the USA, Australia, and Canada [111–114]. For example, stores can allocate more shelf space to display healthy foods, have more refrigerators to store FV, improve the exterior of the store to improving community perception, and assist with promotion and marketing of healthier foods (using shelf labels, call out messages, food and beverage price discounts, placing healthier foods instead of unhealthy foods at eye level or in checkout areas) [111–114]. In the USA, nutrition assistance programs such as SNAP and WIC were reported to increase healthy food access for residents in resource-poor communities [95, 100], and encouraging convenience/corner stores to accept nutrition assistance program benefits may improve healthy food access [106, 108, 109]. Various USA based non-profit organizations, community organizations, and local governments have developed interventions to increase access to healthy foods by modifying existing stores to be healthier food outlets [115].

The present study had several strengths and limitations. To ensure a transparent, reproducible review process and to guide the reporting of results (synthesis), we followed the PRISMA-SCR guidelines. A strict eligibility criterion was followed, and selection and data extraction of studies were done by two reviewers to minimize selection bias. Only published peer-reviewed studies were included whilst grey literature was excluded. The use of peer-reviewed literature may lead to publication bias because studies with null or negative association may not have been published. However, to minimize bias, nine databases were used to search for literature. Restrictions on the publication language is a limitation as articles that were not written in English were potentially excluded. No formal appraisal was conducted since the purpose of a scoping review is to describe evidence, not to assess its quality. The lack of appraisal may have resulted in inclusion of studies with poor methodological quality. The present study included mostly cross-sectional studies therefore we cannot determine a causal relationship between local retail food environment and food choices. Research using longitudinal study designs have been recommended to account for changes in the food environment over time and to improve the quality of evidence [31, 45]. Most studies included in the present review were conducted in the USA, Brazil, and Australia therefore these findings cannot be generalized for other regions. It is recommended that more studies be conducted in European, Asian, and African communities for more evidence on the relationship between local retail food environment and adult food choices.

## Conclusions

The present scoping review found confounding evidence on the relationship between adult food choices and the local retail food environment. Inconclusive findings may be partly due to heterogeneity in measures of food environment exposures. Nonetheless, store types such as supermarkets, large grocery stores and farmers markets, lower in-store food prices and food assistance programs were identified as the main facilitators to healthy food access in resource poor-communities, while high food cost and lack of transportation were identified as the major barriers. Interventions to improve the retail food environment and access to healthy food are mostly based in the USA, Canada, and Australia [116, 117]. Regionally specific interventions to improve healthy food access need to be developed. Evidence on food choices within the context of the retail food environment in countries in Asia and Africa is lacking, and research in these regions are needed to enable the develop of interventions to improve access to healthy food [35, 50].

## Supplementary Information

**Additional file 1.** Preferred Reporting Items for Systematic reviews and Meta-Analyses extension for Scoping Reviews (PRISMA-ScR) Checklist.

**Additional file 2:** **Table S1.** Database search strategies.

**Additional file 3**: **Table S2**. Data extraction form.

## Data Availability

The data supporting the conclusions of this study are included in this published article and its supplementary information files.

## Abbreviations

AHEI: Alternative healthy eating index
ASB: Artificially sweetened beverages
BTG-COMP: Bridging the gap community obesity measures
ESAO-S: Obesogenic environment study food store observation tool
FF: Fast food
FFQ: Food frequency questionnaire
FV: Fruits and vegetables
GIS: Geographic information system
HEI: Healthy eating index
HFSI: Healthy food store index
NEMS: Nutrition environment measures survey
NEMS-C: Nutrition environment measures survey corner store
NEMS-P: Perceived nutrition environment survey
NEMS-R: Nutrition environment measures survey restaurant
PCC: Population, concept and context
PRISMA-ScR: Preferred reporting items for systematic reviews and meta-analyses extension for scoping reviews
SEP: Socioeconomic position
SES: Socioeconomic status
SNAP: Supplemental nutrition assistance program
SSB: Sugar-sweetened beverages
SES: Socioeconomic status
TIABs: Titles and abstracts
WIC: Women, infants, and children
