# Supplementary material for: Adult food choices in association with the local retail food environment and food access in resource-poor communities: a scoping review
Source: BMC Public Health. 2023 Jun 6;23:1083. doi: 10.1186/s12889-023-15996-y (PMC10243040; doi:10.1186/s12889-023-15996-y)
Supplement: Supplementary file 2 — Additional file 2: Table S1. Database search strategies. [file 12889_2023_15996_MOESM2_ESM.docx]

**Table S1. Database search strategies**

| **Database** | **Search terms** | **Number of articles** | **Notes** |
| --- | --- | --- | --- |
| PubMed | Query box  ((((Food choice[Title/Abstract] OR food behaviours[Title/Abstract] OR adult[Title/Abstract] OR food[Title/Abstract] OR fruit[Title/Abstract] OR vegetable[Title/Abstract] OR diet[Title/Abstract] OR nutrition[Title/Abstract] OR processed food[Title/Abstract] OR salty food[Title/Abstract] OR fatty foods[Title/Abstract] OR sugar-sweetened beverages[Title/Abstract] OR fast food[Title/Abstract] OR street food.[Title/Abstract]) AND (Food environment[Title/Abstract] OR nutrition environment[Title/Abstract] OR Local retail food environment[Title/Abstract] OR neighbourhood[Title/Abstract] OR consumer nutrition environment[Title/Abstract] OR community nutrition environment[Title/Abstract] OR food desert[Title/Abstract] OR food swamp[Title/Abstract])) AND (Low income[Title/Abstract] OR low socio-economic status[Title/Abstract] OR disadvantaged[Title/Abstract] OR resource poor[Title/Abstract] OR poor[Title/Abstract] OR poverty[Title/Abstract] OR deprived[Title/Abstract])) AND (Food access[Title/Abstract] OR food availability[Title/Abstract] OR food cost[Title/Abstract] OR food affordability[Title/Abstract] OR food price[Title/Abstract] OR food quality[Title/Abstract])) AND (Food store[Title/Abstract] OR supermarket[Title/Abstract] OR grocery store[Title/Abstract] OR convenience store[Title/Abstract] OR corner store[Title/Abstract] OR fast food[Title/Abstract] OR restaurant[Title/Abstract] OR street vendor[Title/Abstract]) | Search results: 108 | Limiters   - Text Availability- selected Abstract   Filters   - Language-English - Species Human - Age – Young adult, Adult, Middle aged |
|  |  | Updated search results: 4 | Limiters   - Year :2021 – 2022   Filters applied: Abstract, Full text, Young Adult: 19-24 years, Adult: 19-44 years, Middle Aged + Aged: 45+ years, Middle Aged: 45-64 years |
| CINAHL | Boolean phrase  Food choice OR food behaviours OR adult OR food OR fruit OR vegetable OR diet OR nutrition OR processed food OR salty food OR fatty foods OR sugar-sweetened beverages OR fast food OR street food AND Food environment OR nutrition environment OR Local retail food environment OR neighbourhood OR consumer nutrition environment OR community nutrition environment OR food desert OR food swamp AND Low income OR low socio-economic status OR disadvantaged OR resource poor OR poor OR poverty OR deprived AND Food access OR food availability OR food cost OR food affordability OR food price OR food quality AND Food store OR supermarket OR grocery store OR convenience store OR corner store OR fast food OR restaurant OR street vendor | Search results: 121 | Limiters   - Year :2021 – 2022   Filters applied: Abstract, Full text, Young Adult: 19-24 years, Adult: 19-44 years, Middle Aged + Aged: 45+ years, Middle Aged: 45-64 years |
|  |  | Updated search results: 39 | Limiters   - Abstract field - Academic Journals - Abstract Available - Published Date: 20210101-20221231 - English Language - Peer Reviewed |
| EBSCOhost (Academic complete) | Boolean phrase  AB ( Food choice OR food behaviours OR adult OR food OR fruit OR vegetable OR diet OR nutrition OR processed food OR salty food OR fatty foods OR sugar-sweetened beverages OR fast food OR street food ) AND AB ( Food environment OR nutrition environment OR Local retail food environment OR neighbourhood OR consumer nutrition environment OR community nutrition environment OR food desert OR food swamp ) AND AB ( Low income OR low socio-economic status OR disadvantaged OR resource poor OR poor OR poverty OR deprived Food access OR food availability OR food cost OR food affordability OR food price OR food quality ) AND AB ( Food store OR supermarket OR grocery store OR convenience store OR corner store OR fast food OR restaurant OR street vendor ) | Search results 113 | Limiters  • Abstract field  • Scholarly (Peer Reviewed)   - Published Date: 20050701-20101231 - Language: English - Expanders - Apply equivalent subjects |
|  |  | Updated search results: 94 | Limiters   - Abstract field - Scholarly (Peer Reviewed) Journals; Published Date: 20210201-20220331 - Language: English - Expanders - Apply equivalent subjects - Search modes - Boolean/Phrase - Source Types- Academic Journals |
| Green FILE | Boolean phrase  TX ( Food choice OR food behaviors OR adult OR food OR fruit OR vegetable OR diet OR nutrition OR processed food OR salty food OR fatty foods OR sugar-sweetened beverages OR fast food OR street food ) AND TX ( Food environment OR nutrition environment OR Local retail food environment OR neighbourhood OR consumer nutrition environment OR community nutrition environment OR food desert OR food swamp ) AND TX ( Low income OR low socio-economic status OR disadvantaged OR resource poor OR poor OR poverty OR deprived Food access OR food availability OR food cost OR food affordability OR food price OR food quality ) AND TX ( Food store OR supermarket OR grocery store OR convenience store OR corner store OR fast food OR restaurant OR street vendor) | Search results: 27 | Limiters   - Text field - Scholarly (Peer Reviewed) Journals - Publication Date: 20050701-20210131 - Expanders - Apply related words; Apply equivalent subjects |
|  |  | Updated search results: 4 | Limiters   - Scholarly (Peer Reviewed) Journals - Publication Date: 20110101-20211231 - Publication Type: Academic Journal - Expanders - Apply related words; Apply equivalent subjects |
| PsycARTICLES | TX ( Food choice OR food behaviours OR adult OR food OR fruit OR vegetable OR diet OR nutrition OR processed food OR salty food OR fatty foods OR sugar-sweetened beverages OR fast food OR street food ) AND TX ( Food environment OR nutrition environment OR Local retail food environment OR neighbourhood OR consumer nutrition environment OR community nutrition environment OR food desert OR food swamp ) AND TX ( Low income OR low socio-economic status OR disadvantaged OR resource poor OR poor OR poverty OR deprived Food access OR food availability OR food cost OR food affordability OR food price OR food quality ) AND TX ( Food store OR supermarket OR grocery store OR convenience store OR corner store OR fast food OR restaurant OR street vendor ) | Search Results: 137 | Limiters   - Year of Publication: 2011-2021 - Expanders - Apply equivalent subjects - Search modes - Boolean/Phrase |
|  |  | Updated search results :15 | Limiters   - Text field - Scholarly (Peer Reviewed) Journals - Published date 20210201-20220331 - Expanders - Apply equivalent subjects |
| Social Science Research Network | Food choice AND Food environment AND Food access | Search results: 15 | Limiters  • Title & Abstract & keywords only   - Network selection ALL SSRN - Date: 2005 -2021 |
|  |  | Updated search results: 1 | Limiters   - Title & Abstract & keywords only - Network selection ALL SSRN - Date: 2021   No references could be saved to ENDNOTE |
| SCOPUS | Food choice AND Food environment AND Low Income AND Food access | Search results: 64 | Limiters   - Date: 2005- 2021 |
|  |  | Updated search results:12 | Limiters   - Date: 2021-2022 |
| Science Direct  Research articles | Food choice AND Adult AND Local retail food environment AND Low income AND Food access AND Food stores | Search results: 721 | Limiters   - Filter 2011 -2021 - Article type: review articles & research articles |
|  |  | Updated search results :120 | Limiters   - Filter 2021- 2022 - Article type: review articles & research articles |
| Web of Science  Basic Search | TOPIC: (Food choice AND Food environment AND Low income AND Food access AND Food stores) | Search results: 826 | Limiters  • Publication years :2005 - 2021 |
|  |  | Updated search result 5 | Limiters   - Publication years :2021 |
