# Supplementary material for: Adult food choices in association with the local retail food environment and food access in resource-poor communities: a scoping review
Source: BMC Public Health. 2023 Jun 6;23:1083. doi: 10.1186/s12889-023-15996-y (PMC10243040; doi:10.1186/s12889-023-15996-y)
Supplement: Supplementary file 3 — Additional file 3: Table S2. Data extraction form. [file 12889_2023_15996_MOESM3_ESM.docx]

**Table S2**. **Data extraction form**

| Scoping review - Data extraction form | | |
| --- | --- | --- |
| **Data to be extracted** | **Notes for reviewer** |  |
| Name of reviewer | *Tick* | **Reviewer 1 Reviewer 2** |
| Date | *DD/MM/YYYY* |  |
| Authors |  |  |
| Title of study |  |  |
| Year of Publication |  |  |
| Aim/objective of the study |  |  |
| Study Area | *Location/Country* |  |
| Study setting | *What is the geographical setting of study?* | Urban Rural  Semi-urban Semi-rural |
| Study Participants | - *Adults (Number, Age & Gender, Ethnicity)* - *No. of communities* - *No. of Food stores* |  |
| Sampling method | *Sampling type* | Random Systematic Cluster Stratified Purposive Quota  Snowball Convenience Other: |
| Study design | *Primary research or Review?* | Cross-sectional Cohort  Case-control Other: |
| Data collection | *Methods* | Quantitative Mixed method  Qualitative Other: |
| Measurement tools (Quantitative & Mixed method) | *Measurement tools for food choice /food environment/food access e.g score, index, GIS based, checklist/survey etc.* |  |
| Measurement tools (Qualitative) |  | Interviews Focus group  Participatory Observation |
| Data analysis | *How are data analysed?* |  |
| Reported Outcomes | *Study findings relevant to study objectives.*  *Association between adult food choices & the food environment/food access.* |  |
| Most relative findings  *Findings as relates to food choices and healthy diet measured by fruit and vegetable intake, various food group intake, intake of salty and fatty foods, sugar-sweetened beverage intake, fast-food intake, diet quality, energy and micronutrient intake, healthy diet score versus unhealthy diet scores* | Themes (Qualitative) |  |
|  | Conclusions |  |
|  | Opinions (Review)  *What this person argues?* |  |
| Facilitators | *Describe the factors that enable healthy food choices and food access in the local retail food environment*. |  |
| Barriers | *Describe the factors that hinder healthy food choices and food access in the local retail food environment.* |  |
| Decision | *Inclusion*  *Should this study be included in the final review?* | Yes No Unclear |
| Reason for Exclusion |  |  |
